# Early versus late distant metastasis and adjuvant chemotherapy alone *versus* both radiotherapy and chemotherapy in molecular apocrine breast cancer

**DOI:** 10.18632/oncotarget.10211

**Published:** 2016-06-21

**Authors:** Xiaozhen Liu, Yang Yang, Xiaolong Feng, Honghong Shen, Jian Liu, Xia Liu, Yun Niu

**Affiliations:** ^1^ Key Laboratory of Breast Cancer Prevention and Therapy (Ministry of Education), Tianjin Medical University Cancer Institute and Hospital, National Clinical Research Center for Cancer, Key Laboratory of Cancer Prevention and Therapy, Tianjin, China; ^2^ Department of Oncology, General Hospital of Tianjin Medical University, Tianjin, China

**Keywords:** androgen receptor, distant metastasis, molecular apocrine breast cancer, radiotherapy and chemotherapy, survival rate, Pathology Section

## Abstract

As a new subtype of breast cancer, molecular apocrine breast cancer (MABC) is estrogen receptor (ER) and progesterone receptor (PR) negative expression, but androgen receptor (AR) positive expression. The prognostic significance and clinical biological behavior of MABC have remained unclear up to now. This study aimed to analysis the distant metastasis behavior and response to adjuvant radiotherapy and chemotherapy of MABC subgroup. The report showed that there were significant differences between early and late distant metastasizing tumors with respect to Ki67, epidermal growth factor receptor 2 (HER2) and vascular endothelial growth factor (VEGF) expressions by a retrospective analysis consisting of 410 invasive breast cancer patients, which included 205 MABC and 205 nonMABC cases. MABC subgroup metastasized earlier than nonMABC subgroup, and MABC showed a tendency for a higher metastasis rate in lung, liver and brain, but lower in bone. HER2-positive or VEGF-positive tumors were more inclined to develop bone metastasis within MABC subgroup. The survival rate was superior for patients undergone both adjuvant radiotherapy and chemotherapy than those undergone chemotherapy alone in nonMABC subgroup, but there was no significant difference in MABC subgroup. Our data suggested that MABC subgroup seemed to develop distant metastasis earlier than nonMABC subgroup, and patients with MABC indicated poor prognosis. This study might also provide a foundation for helping patients receive reasonable treatments according to molecular subtype.

## INTRODUCTION

In addition to estrogen receptor (ER) and progesterone receptor (PR), there is obvious evidence that the androgen signaling pathway may also play a critical role in breast cancer [1, 2]. Depending on the breast cancer molecular subtype, androgen receptor (AR) and androgen signaling may have either tumor suppressive or oncogenic role on breast cancer growth. The association between AR expression and favorable outcome in ER positive breast cancer had been verified in various studies [3, 4]. Tsang et al [5] showed that in ER positive breast cancers, AR expression was associated with a lower grade disease and a better prognosis, whereas in ER negative breast cancers, AR appeared to be capable of mediating proliferation and thus acting an oncogenic driver. However, the role of AR expression in ER negative breast cancer has not reached consensus up to now. In 2005, Farmer et al [6] named ER negative and AR positive tumors as molecular apocrine breast cancer (MABC), while these lesions did not meet the strict histopathological criteria for diagnosis as classical apocrine carcinomas. Then in 2013, Lehmann-Che et al [7] initially confirmed a group of breast cancer samples by a molecular apocrine qRT-PCR signature and then performed immunohistochemistry, and they reported that only 4 morphological apocrine tumors among 58 molecular apocrine cases, which suggested that MABC subgroup could in fact be much broader than initially reported by Farmer et al. In 2014, Lakis's [8] study subtyped tumors into luminal, molecular apocrine (ER-/PR-/AR+) and receptor-negative, and it had proved that AR-related subtype of breast cancer might be prognostic and serve for selecting optimal treatment combinations. However, Cha et al [9] found that there were no significant differences in patient prognosis between MABC and other types of breast cancer. Therefore, the prognostic significance and clinical biological behavior of MABC were needed to be better understood.

Although the primary tumor growth can be prevented by surgery, adjuvant radiotherapy and chemotherapy, most breast cancer deaths are usually related to distant organ metastasis which is not very effective in preventing, even more is considered to be essentially incurable. As breast cancer causes mortality mainly by metastasizing to a variety of vital organs, such as bone, lung, brain and liver, it is always characterized by heterogeneity. Furthermore, the metastatic spread of breast cancer is often organ-specificity [10]. Thus the probability to assess the metastasis organs for different breast cancer molecular subtypes is quite useful in the treatment. However, this has not been well defined yet.

Therefore, in the current study, we emphasized analyzing the characteristics of a unique breast cancer molecular subgroup, MABC, which is characterized by ER and PR negative, but AR positive. To achieve this, the distant metastasis behavior and response to adjuvant radiotherapy and chemotherapy were investigated in patients with MABC and nonMABC. It was hypothesized that the results may assess the organ of metastasis in the development of MABC. Additionally, this study aimed to identify reasonable treatments that may be useful to improve breast cancer patients' quality of life during the course of the disease.

## RESULTS

### Characteristics of patients and tumors

**Figure 1:**
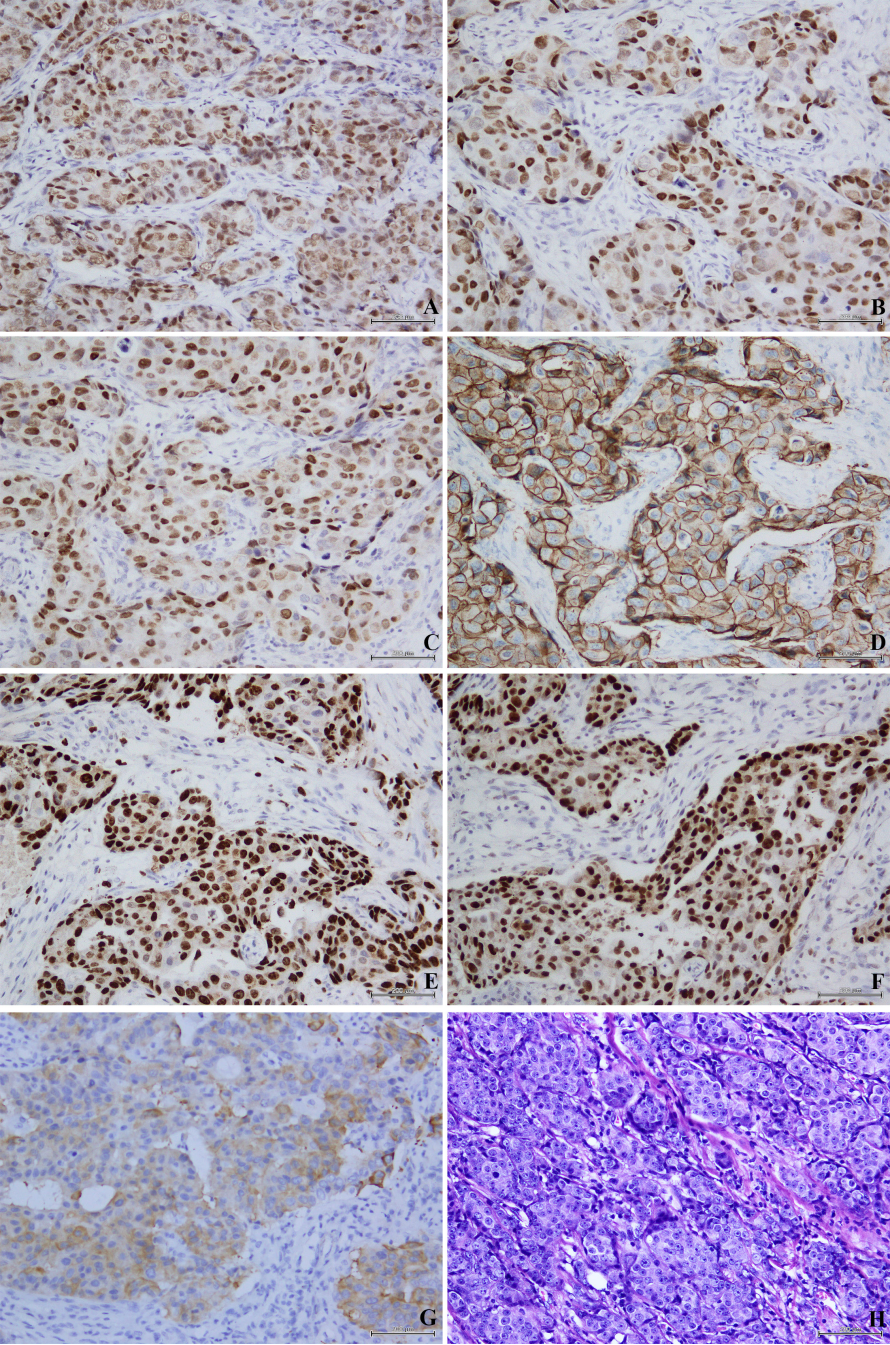
Location of immunohistochemical staining of each protein marker in invasive breast cancer tissues Immunohistochemical staining of estrogen receptor (ER) revealed nuclear staining **A.**, progesterone receptor (PR) revealed nuclear staining **B.**, androgen receptor (AR) revealed nuclear staining **C.**, epidermal growth factor receptor 2 (HER2) revealed cytomembrane staining **D.**, Ki67 revealed nuclear staining **E.**, p53 revealed nuclear staining **F.** and vascular endothelial growth factor (VEGF) revealed cytoplasm staining **G.**; HE staining **H.** of invasive breast cancer tissues. Original magnification ×200.

**Figure 2:**
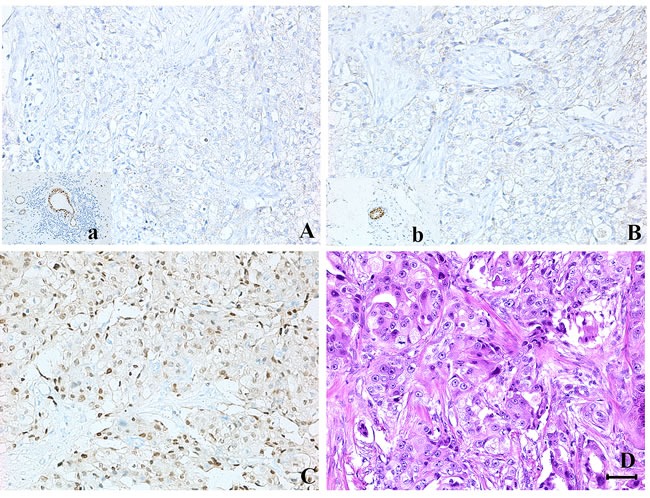
A tumor classified as carcinomas with apocrine differentiation, which had the immunohistochemical characteristics of molecular apocrine breast cancer (MABC) **A.**, **B.** and **C.** Immunohistochemical staining of ER (negative), PR (negative) and AR (positive) in carcinomas with apocrine differentiation. **D.** HE staining of carcinomas with apocrine differentiation. a and b. Positive controls for ER and PR. Original magnification ×200. (Bar = 80μm)

The study randomly selected 1000 patients with invasive breast carcinoma. According to the expression of ER, PR and AR, this study contained 205 MABC (ER-/PR-/AR+) and 795 nonMABC patients, and then we randomly selected 205 nonMABC patients from these 795 samples. All of these 410 cases were tested by immunohistochemical staining for epidermal growth factor receptor 2 (HER2), Ki67, p53 and vascular endothelial growth factor (VEGF). Location of immunohistochemical staining of each protein marker in breast cancer tissues was illustrated in Figure 1. Figure 2 showed a tumor classified as carcinomas with apocrine differentiation, which had the immunohistochemical characteristics of MABC (ER-/PR-/AR+).

Details of the patients and tumors were shown in Supplementary table 1. Patients in this cohort were female ranging in age from 31 to 78 years at diagnosis, and median age was 53 years. Results showed that most of the MABC patients (56.1%, 115/205) were pre-/perimenopausal *versus* 39.5% (81/205) of the nonMABC patients (χ^2^ = 11.300, *P* = 0.001). The majority of the tumors were classified as invasive carcinoma of no specific type (NST), and the others included invasive lobular carcinoma (ILC), carcinomas with apocrine differentiation, invasive micropapillary carcinoma (IMPC), invasive papillary carcinoma (IPC), carcinomas with medullary features and other invasive carcinomas. Among 410 enrolled patients, 78 cases developed distant metastasis, including 48 MABC and 30 nonMABC cases, and the median time for first distant metastasis was 28 months (range, 5-111 months) and 61 months (range, 5-106 months), respectively.

### Characteristics of early *versus* late distant metastasis

Using the cut-off point of 5 years, 54 (69.2%) cases were recorded as early distant metastasizing tumors, while 24 (30.8%) were the late ones among 78 distant metastasizing tumors. Furthermore, there were 41 early and 7 late distant metastasizing tumors in MABC subgroup. While in nonMABC subgroup, 13 were recorded as early distant metastasizing tumors, and 17 were the late ones. Results showed that MABC subgroup metastasized earlier than nonMABC subgroup (*P* < 0.001, Table 1). As MABC includes tumors with ER−/PR−/HER2−/AR+ and ER−/PR−/HER2+/AR+ and others all belonged to the nonMABC subgroup, we then analyzed the difference of time to distant metastasis in ER−/PR−/HER2−/AR+ and ER−/PR−/HER2−/AR− subgroups, as well as in ER−/PR−/HER2+/AR+ and ER−/PR−/HER2+/AR− subgroups. We then found that tumors with ER−/PR−/HER2−/AR+ and ER−/PR−/HER2+/AR+ metastasized earlier than the corresponding comparative groups (*P* = 0.018, *P* = 0.030, Table 1). The association between clinicopathological and biological variables and early *versus* late distant metastasis was shown in Table 2. It indicated that there were significant differences between early and late distant metastasizing tumors with respect to histological grade (*P* = 0.003), tumor stage (*P* = 0.019), lymph node metastasis (*P* = 0.035) and TNM stage (*P* = 0.017). It showed that higher histological grade, tumor stage, TNM stage and more lymph node metastasis tumors metastasized earlier than the others. The early distant metastasizing tumors seemed to have higher proliferative ability by Ki67 labeling (*P* = 0.044), and exhibited higher incidence of HER2 and VEGF positivity expression than the late ones (*P* = 0.033, *P* = 0.004). But time to distant metastasis had no differences among patients' age, menopausal status, and p53 expression (*P* = 0.688, *P* = 0.909, *P* = 1.000).

**Table 1:** Correlation between time to distant metastasis and different molecular subtypes

Molecular subtypes	≤5 years (*N*= 54)	>5 years (*N*= 24)	χ^2^	*P* value
MABC	41	7	15.349	<0.001
nonMABC	13	17		
ER-/PR-/HER2-/AR+	21	5	5.640	0.018
ER-/PR-/HER2-/AR-	5	7		
ER-/PR-/HER2+/AR+	20	2	4.693	0.030
ER-/PR-/HER2+/AR-	5	4		

AR, androgen receptor; ER, estrogen receptor; HER2, epidermal growth factor receptor 2; MABC, molecular apocrine breast cancer; PR, progesterone receptor.

*P* values were calculated by Chi-square test.

**Table 2:** Correlation between time to distant metastasis and clinicopathological characteristics

Clinicopathological and biological characteristics	Total cases (*N*= 78)	≤5 years %(*N*= 54)	>5 years %(*N*= 24)	χ^2^	*P*value
Age (years)					
<35	1	100.0 (1)	0.0 (0)	0.747	0.688
35-49	39	69.2 (27)	30.8 (12)		
>49	38	68.4(26)	31.6(12)		
Menopausal status					
yes	35	68.6(24)	31.4(11)	0.013	0.909
no	43	69.8(30)	30.2(13)		
Histological grade					
G1	5	20.0 (1)	80.0 (4)	11.729	0.003
G2	34	58.8 (20)	41.2 (14)		
G3	39	84.6 (33)	15.4 (6)		
Tumor stage					
T1	20	65.0 (13)	35.0 (7)	7.939	0.019
T2	41	61.0 (25)	39.0 (16)		
T3	17	94.1 (16)	5.9 (1)		
Lymph node metastasis					
negative	23	52.2 (12)	47.8 (11)	4.455	0.035
positive	55	76.4 (42)	23.6 (13)		
TNM stage					
I	5	20.0(1)	80.0 (4)	8.159	0.017
II	44	65.9 (29)	34.1 (15)		
III	29	82.8 (24)	17.2 (5)		
HER2					
negative	48	60.4 (29)	39.6 (19)	4.552	0.033
positive	30	83.3 (25)	16.7 (5)		
Ki67					
<20%	7	28.6 (2)	71.4 (5)	4.055	0.044
≥20%	71	73.2 (52)	26.8 (19)		
p53					
negative	52	69.2 (36)	30.8 (16)	0.000	1.000
positive	26	69.2 (18)	30.8(8)		
VEGF					
negative	43	55.8 (24)	44.2 (19)	8.098	0.004
positive	35	85.7 (30)	14.3 (5)		

VEGF, vascular endothelial growth factor.

*P* values were calculated by Chi-square test.

### Site of distant metastasis

The organs of distant metastasis were described in Table 3. Among the 78 patients developed distant metastasis, 27 cases developed multiple metastases during the course of follow-up, including 16 MABC and 11 nonMABC cases. Bone was the most common site for metastasis (50 %) followed by lung (34.6%), liver (26.9%), brain (16.7 %) and kidney (3.8 %), respectively. MABC showed a tendency for a higher metastasis rate in lung, liver and brain than nonMABC subgroup (*P* = 0.010, *P* = 0.044, *P* = 0.048), but lower metastasis rate in bone (*P* = 0.012).

**Table 3:** Different metastasis organs between MABC and non-MABC subgroups

Metastasis organ	Total cases	MABC %(*N*= 205)	non-MABC %(*N*= 205)	χ^2^	*P* value
bone					
yes	39	30.8 (12)	69.2 (27)	6.376	0.012
no	371	52.0 (193)	48.0(178)		
lung					
yes	27	74.1(20)	25.9(7)	6.701	0.010
no	383	48.3(185)	51.7(198)		
liver					
yes	21	71.4 (15)	28.6 (6)	4.065	0.044
no	389	48.8 (190)	51.2 (199)		
brain					
yes	13	76.9 (10)	23.1 (3)	3.893	0.048
no	397	49.1 (195)	50.9 (202)		
Kidney*					
yes	3	33.3(1)	66.7(2)	-	-
no	407	50.1(204)	49.9(203)		

* The number was so small that we did not calculate the *P* value.

*P* values were calculated by Chi-square test.

To better understand the behavior of MABC, we then divided MABC into 136 HER2-negative cases and 69 HER2-positive cases, since MABC subgroup included tumors with ER-/PR-/HER2- (triple-negative breast cancer, TNBC) and ER-/PR-/HER2+ (HER2-overexpression). Results showed that there were 9, 8, 4 and 5 cases developed bone, lung, liver and brain metastasis, respectively, in HER2-positive MABC subgroup. While in HER2-negative MABC subgroup, the corresponding cases were 3, 12, 11 and 5, respectively. Furthermore, there was one patient developed kidney metastasis in HER2-negative MABC subgroup. Notably, significant difference was found only between bone metastasis and HER2 status within MABC patients (*P* = 0.002, Table 4).

**Table 4:** Effects of HER2 and VEGF status upon the site of distant metastasis within MABC subgroup

Metastasis organ	Total cases	HER2		χ^2^	*P* value	VEGF		χ^2^	*P* value
		Positive %(*N*= 69)	Negative %(*N*= 136)			Positive (*N*= 66)	Negative (*N*= 139)		
bone									
yes	12	75.0 (9)	25.0 (3)	9.145	0.002	75.0 (9)	25.0 (3)	9.871	0.002
no	193	31.1 (60)	68.9 (133)			29.5 (57)	70.5 (136)		
lung									
yes	20	40.0 (8)	60.0 (12)	0.399	0.528	40.0 (8)	60.0 (12)	0.618	0.432
no	185	33.0 (61)	67.0 (124)			31.4 (58)	68.6 (127)		
liver									
yes	15	26.7 (4)	73.3 (11)	0.367	0.544	53.3 (8)	46.7 (7)	3.313	0.069
no	190	34.2 (65)	65.8 (125)			30.5 (58)	69.5 (132)		
brain									
yes	10	50.0 (5)	50.0 (5)	1.191	0.275	30.0 (3)	70.0 (7)	0.023	0.878
no	195	32.8 (64)	67.2 (131)			32.3 (63)	67.7 (132)		
Kidney*									
yes	1	0.0 (0)	100.0 (1)	-	-	100.0 (1)	0.0 (0)	-	-
no	204	33.8 (69)	66.2 (135)			31.9 (65)	68.1 (139)		

* The number was so small that we did not calculate the *P* value.

*P* values were calculated by Chi-square test.

**Figure 3:**
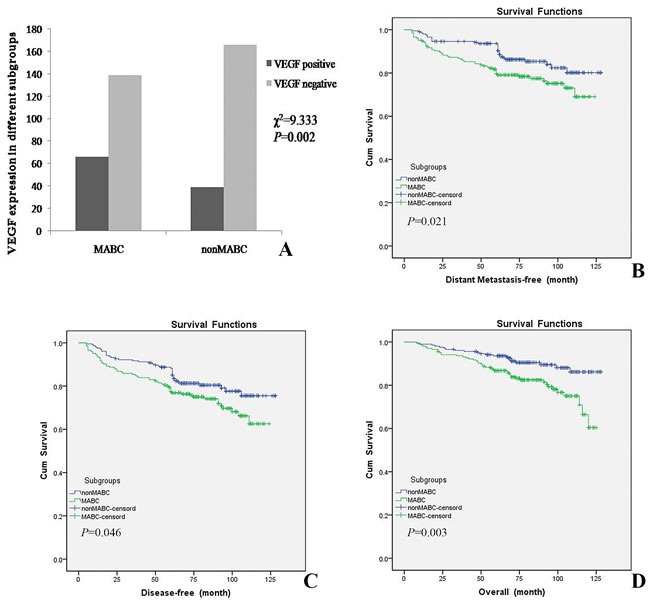
The expression of VEGF was significantly different between MABC and nonMABC subgroups, and patients with MABC indicated poor prognosis than nonMABC subgroup **A.** There was significant difference between VEGF positive expression and MABC subgroup (χ^2^ = 9.333, *P* = 0.002). **B.**, **C.** and **D.**. Distant metastasis-free survival (DMFS), disease-free survival (DFS) and overall survival (OS) curves of 410 patients were generated according to the Kaplan-Meier method.

VEGF was one of major proangiogenic factors, and we then also analyzed whether VEGF played a critical role in the site of distant metastasis within MABC subgroup. There was significant difference between VEGF positive expression and MABC subgroup (χ^2^ = 9.333, *P* = 0.002, Figure 3A). In 66 VEGF-positive MABC subgroup, 9 patients developed bone metastasis, while 8, 8, 3 and 1 patients developed lung, liver, brain and kidney metastasis, respectively. In 139 VEGF-negative MABC subgroup, there were 3, 12, 7 and 7 patients developed bone, lung, liver and brain metastasis, respectively. Interestingly, only bone metastasis was influenced by VEGF expression, and VEGF positive expression tumors were more inclined to develop bone metastasis (*P* = 0.002, Table 4).

### Survival analysis

The distant metastasis-free survival (DMFS) differed significantly between MABC and nonMABC subgroups, and the DMFS of the MABC subgroup was worse than that of the nonMABC subgroup (*P* = 0.021, Figure 3B). The probability of 5-year DMFS for the MABC and nonMABC subgroups was 80.0% and 93.7% and for 10-year DMFS was 76.6% and 85.4%, respectively. The probability of 5-year disease-free survival (DFS) for the MABC and nonMABC subgroups was 77.6% and 88.3% and that of 10-year DFS was 71.7% and 80.0%, respectively. Similarly, the DFS for the MABC subgroup was significantly worse than that of the nonMABC subgroup (*P* = 0.046, Figure 3C). The 5-year and 10-year overall survival (OS) rates were 87.3% and 79.0%, respectively, for MABC, and 94.1% and 89.8%, respectively, for nonMABC, with significant statistical differences (*P* = 0.003, Figure 3D).

**Figure 4:**
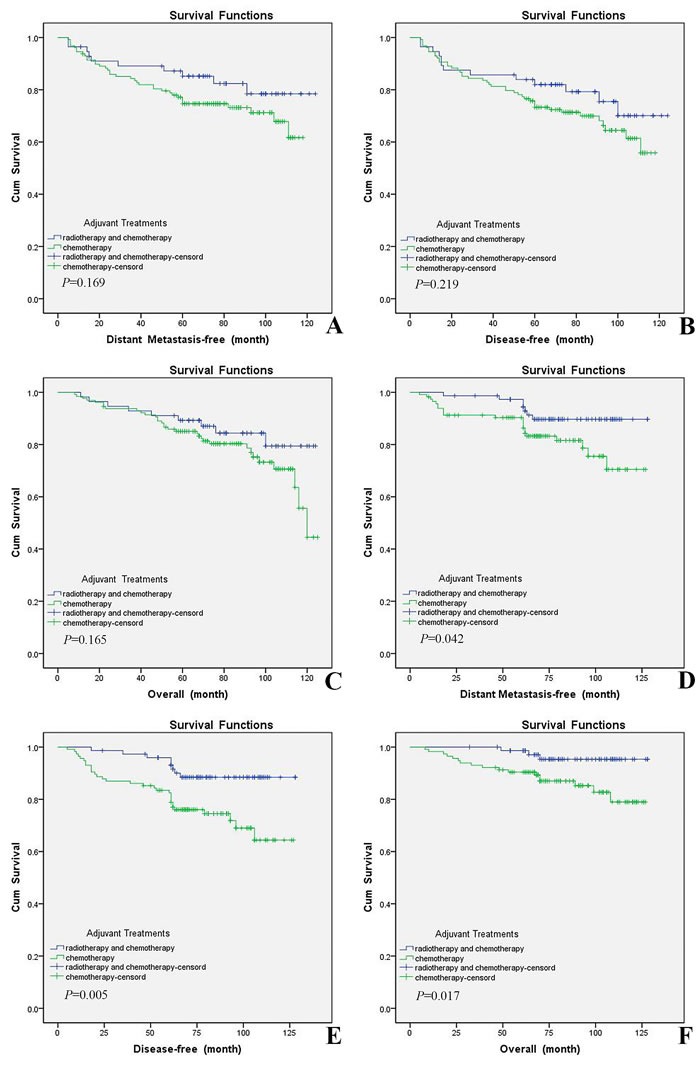
Patients undergone both adjuvant radiotherapy and chemotherapy had relative better outcome than those undergone chemotherapy alone within nonMABC subgroup, but there was no significant difference in MABC subgroup **A.**, **B.** and **C.**. DMFS, DFS and OS curves of 184 MABC patients undergone both radiotherapy and chemotherapy or chemotherapy alone were generated according to the Kaplan-Meier method. **D.**, **E.** and **F.**. DMFS, DFS and OS curves of 190 nonMABC patients undergone both radiotherapy and chemotherapy or chemotherapy alone were generated according to the Kaplan-Meier method.

In MABC subgroup, 128 (62.4%) had undergone chemotherapy alone, and 56 (27.3%) had undergone both radiotherapy and chemotherapy. In addition, in nonMABC subgroup, 115 (56.1%) had undergone chemotherapy alone, and 75 (36.6%) had undergone both radiotherapy and chemotherapy. There were no significant differences for the DMFS, DFS or OS between MABC patients who had undergone chemotherapy alone and undergone both radiotherapy and chemotherapy (*P* = 0.169, *P* = 0.219, *P* = 0.165; Figure 4A, 4B and 4C). However, in nonMABC subgroup, the DMFS was superior for women who were adherent to both radiotherapy and chemotherapy than those who undergone chemotherapy alone (*P* = 0.042; Figure 4D). It seemed that the DFS and OS were also superior for patients who accepted both radiotherapy and chemotherapy, and differences reached statistical significance (*P* = 0.005, *P* = 0.017; Figure 4E and 4F).

## DISCUSSION

We have previously stated that MABC was associated with a serious of increased risk of malignant breast cancer, including higher histological grade, tumor stage, and pTNM stage [11]. Our findings from this study supplemented the conclusion that MABC tumors metastasized earlier than nonMABC. Similar to Huang et al [12], pre-/perimenopausal women were at increased risk of developing breast tumors negative for ER and PR. Results in the current study showed that pre-/perimenopausal patients were at increased risk of developing breast tumors not only negative for ER and PR, but also positive for AR. In agreement with published literature [13], our data clearly indicated that hormone receptor negative breast cancer (ER-/PR-) patients metastasized earlier than hormone receptor positive patients. In addition, the results also showed that AR positive expression tumors metastasized earlier than AR negative ones in hormone receptor negative patients. This might due to the fact that most of the early metastasizing tumors were ER and PR negative expression, and AR appeared to be an oncogenic driver in ER-negative tumors [5]. Ki67 was a proliferative activity index and an established prognostic biomarker of breast cancer, and this study found that only minority (2.6 %) of the early distant metastasizing tumors and 9.0% of all the distant metastasizing tumors expressed lower Ki67 (< 20%). This would be interpreted as Ki67 higher expression tumors were more inclined to develop distant metastasis earlier.

In the treatment of breast cancer patients, the major pattern of failure after surgery was distant metastasis, and an early assessment of metastatic risk was important in different molecular breast cancer therapy. Bone is the most common site for distant metastasis and is of particular clinical importance in breast cancer patients. It has been shown that hormone receptor positive breast cancers have a greater tendency to develop bone metastasis [14-16]. Kennecke et al [17] stated that bone was the most common metastatic site in all subtypes except basal-like breast cancer. In addition, researchers [17-21] have also found that receptor negative breast cancer showed increased incidence of visceral and cerebral distant metastasis. However, Koo et al [15] stated thatlung metastasis showed all of three subtypes (triple-negative type, HER-2 type, ER+ or PR+/HER-2- type) in similar proportions. This study found that nonMABC patients had a greater tendency to develop bone metastasis, and MABC showed a tendency for a higher metastasis rate in lung, liver and brain. Results also showed that the median time for first metastasis was 28 months and 61 months in MABC and nonMABC subgroups, respectively. Moreover, 16 MABC and 11 nonMABC cases developed multiple metastases during the course of follow-up. All these results revealed that receptor negative breast cancer patients had a high risk to develop advanced diseases, and AR expression could not reverse the progression of disease in hormone receptor negative breast cancer. AR might also influence the site of distant metastasis in breast cancer, so more studies should be carried out to confirm this hypothesis.

MABC has a distinct hormone receptor profile, being ER and PR negative, but AR positive, and HER2 either positive or negative expression. So the effect of HER2 expression on distant metastasis was analyzed in the current study. In agreement with previously published data, HER2 positivity was not identified as a strong predictive factor for site specific metastasis [13], except for bone metastasis. However, some researchers have found that HER2-enriched tumors were associated with a significantly higher rate of brain, liver, and lung metastases [17, 22]. These differences might due to the fact that AR expression could regulate the behavior of breast cancer, especially tumors with ER and PR negative expression. Therefore, HER2 status might regulate the site of distant metastasis partly dependent on AR positive expression in tumors with ER and PR negative expression.

The density of microvessels of tumorigenesis could reflect patient's prognosis, and VEGF is a key mediator of proangiogenic factors. VEGF promotes vascular endothelial growth and mediates vessel permeability, thus facilitating tumor progression and metastatic spread [23], and high protein levels of VEGF seemed to be associated with poor prognosis in breast cancer patients [24]. In the current study, high positive rate of VEGF expression was detected in MABC subgroup and VEGF positive expression patients developed distant metastasis earlier than those with VEGF negative expression. In addition, VEGF was observed to be upregulated in bone metastases compared with those in the lung, liver or brain in MABC subgroup. The results of the present study confirmed that high expression of VEGF protein was more common in patients with MABC and was usually associated with a higher incidence of distant metastasis, especially bone metastasis.

It has been indicated that in patients with ER-positive tumor, AR expression was associated with a better outcome [25, 26]. The published literature has yielded extremely controversial findings regarding the potential prognostic role of AR expression in hormone receptor negative breast cancer. Some researchers [27, 28] reported that AR positivity was not associated with DFS or OS, but Mrklic et al [28] found that AR expression correlated inversely with higher mitotic score, clinical stage, histological grade, and Ki-67 proliferation index. Agoff et al [29] highlighted an association of AR expression with better DFS, while Hu et al [3] reported that AR expression was an unfavorable prognostic factor. In the present study, we found that AR expression was not only associated with worse DFS and OS, but also associated with worse DMFS in receptor negative breast cancer. However, further studies adopting more samples should be adopted to validate the present findings and uncover potential underlying mechanisms and molecular pathways.

Studies have confirmed that the use of chemotherapy was associated with an improved overall survival rate, and no difference was observed between the various chemotherapy regimens used [30-32]. Bulut et al [33] stated that patients with triple negative breast cancer had similar survival with radiotherapy and chemotherapy. This study assessed survival rate between patients who had undergone both radiotherapy and chemotherapy and those undergone chemotherapy alone in different molecular subgroups. Results indicated that MABC patients might receive chemotherapy only, as no difference was found between patients who had undergone chemotherapy alone and undergone both radiotherapy and chemotherapy. On the contrary, survival rate was superior for patients undergone both radiotherapy and chemotherapy in nonMABC subgroup. However, we did not take into consideration the endocrine therapy or trastuzumab treatment, which could influence the outcome of ER-positive or HER2-positive breast cancer patients. Further studies on larger cohorts of patients are required to assess the role of molecular subtype in breast cancer treatment, and more factors affected patients' prognosis should also be taken into account.

In conclusion, these findings led to a new understanding of MABC and confirmed that molecular subtypes were strongly related to distant metastasis, in terms of site-specific metastasis, early/late metastasis and survival outcomes. NonMABC patients have a tendency to develop bone metastasis and they have better survival outcomes compared to MABC subgroup with a tendency of developing viscera and brain metastasis. In addition, the outcome of MABC subgroup was similar between those undergone both radiotherapy and chemotherapy and those undergone chemotherapy alone, but the outcome was superior for patients undergone radiotherapy and chemotherapy in nonMABC subgroup. This study also clarified that targeting AR would present an attractive treatment option for MABC cases, and these results were of help in choices for treatment in individual breast cancer patients.

## MATERIALS AND METHODS

### Patients and grouped

The study randomly selected 1000 patients with invasive breast carcinoma enrolled between January 2004 and December 2005. The cases were registered in the archives of the Department of Breast Cancer Pathology and Research Laboratory, Tianjin Medical University Instituted and Cancer Hospital, Tianjin, China. This study was reviewed and approved by the Institutional Ethic Committee of Tianjin Medical University Instituted and Cancer Hospital. Informed consent was obtained from all the patients before their surgery and the examination of the specimens. Patients with a previous cancer during the last 10 years or patients with bilateral primary breast cancers were excluded. All of these cases had not received preoperative treatments and their clinicopathologic date was available. First of all, all of these cases were tested by immunohistochemical staining for ER, PR and AR, and samples were divided into MABC (ER-/PR-/AR+) and nonMABC subgroups. As the quantity of MABC was much smaller than nonMABC, we then randomly selected a sample among nonMABC which had the same number with MABC subgroup. Then all of these selected cases were tested by immunohistochemical staining for HER2, Ki67, p53 and VEGF.

### Immunohistochemical assay and evaluation of the staining

Immunohistochemistry was carried out as previously reported [34]. The slides were immunostained using primary antibodies against ER (SP1, 1: 200 dilution; ZETA), PR (SP2, 1: 200 dilution; ZETA), AR (AR441, 1: 100 dilution; LabVision), HER2 (CB11, 1: 100 dilution; Invitrogen), Ki67 (K-2, 1:100 dilution; Invitrogen), p53 (SP5, 1:100 dilution; Invitrogen) and VEGF (ZA-0580, 1:50 dilution; ZSGB). Sections of normal breast tissue were processed simultaneously and served as positive controls for ER and PR. Similarly, HER2, Ki67, p53 and AR positive breast cancer tissues were used as positive controls for HER2, Ki67, p53 and AR, respectively. In addition, normal goat serum substituted primary antibodies as negative controls.

The immunostaining was scored by two senior pathologists, who were blinded to patients' clinicopathologic characteristics and outcomes. For each antibody, the location of immunoreactivity, percentage of stained cells, and intensity were determined. ER and PR were considered positive if nuclear staining was present in more than 1% of the tumor cells; HER2 was considered positive when there was a strong whole membrane staining in > 10% of the tumor cells; Ki67 was expressed as percentage of positive cells (strong nuclear staining), with a threshold of 20% or above being considered high; Tumors with 10% or greater nuclear positivity were considered to be positive for p53 and AR; As for VEGF, at least 10% of cells (cytoplasm) needed to be stained to be considered positive.

### Follow-up

All the patients had been treated according to modern guidelines, including operation, the use of adjuvant chemotherapy, radiotherapy, and endocrine and targeted therapy. We retrospectively reviewed 128 months follow-up data. The follow-up contacts were carried out at 3-month intervals over the first year, 6-month intervals during the second year, and at 12-month intervals thereafter. The methods included clinical physical examination, x-ray, ultrasound techniques, CT as well as puncture biopsy verification to suspected metastatic lesions. The follow-up scheme was the same for all patients.

### Statistic analyses

Statistical analysis was carried out using SPSS 19.0 statistical software. Differences of continuous variables between MABC and nonMABC subgroups, as well as early and late distant metastasis tumors, were evaluated by the Chi-square test. DMFS, DFS and OS curves were generated according to the Kaplan-Meier method. The differences between the curves were assessed using the log-rank test. *P* < 0.05 was considered statistically significant.

## SUPPLEMENTARY MATERIALS TABLE


