# Supplementary material for: Early versus late distant metastasis and adjuvant chemotherapy alone versus both radiotherapy and chemotherapy in molecular apocrine breast cancer
Source: Oncotarget. 2016 Jun 21;7(31):48905–17. doi: 10.18632/oncotarget.10211 (PMC5226479; doi:10.18632/oncotarget.10211)
Supplement: Supplementary file 1 [file oncotarget-07-48905-s001.pdf]

Early *versus* late distant metastasis and adjuvant chemotherapy alone *versus* both radiotherapy and chemotherapy in molecular apocrine breast cancer

Supplementary Material

Supplementary Table 1: Details of the cohort patients and tumors

Clinicopathological and biological characteristics	Total cases (N=410)	MABC (N=205)	nonMABC (N=205)
Age (years)			
<35	6	4	2
35-49	192	85	107
>49	212	116	96
Menopausal status			
yes	214	90	124
no	196	115	81
Histological grade			
G1	15	3	12
G2	253	107	146
G3	142	95	47
Tumor stage			
T1	132	53	79
T2	228	121	107
T3	50	31	19
Lymph node metastasis			
negative	205	94	111
positive	205	111	94
TNM stage			
Ⅰ	77	28	49
Ⅱ	233	119	114
Ⅲ	100	58	42
ER			
negative	290	205	85
positive	120	0	120
PR			

negative	325	205	120
positive	85	0	85
HER2			
negative	285	136	149
positive	125	69	56
Ki67			
<20%	131	41	90
⩾20%	279	164	115
P53			
negative	310	148	162
positive	100	57	43
VEGF			
negative	305	139	166
positive	105	66	39
AR			
negative	100	0	100
positive	310	205	105
Histological type			
invasive carcinoma of no specific type	357	185	172
invasive lobular carcinoma	7	2	5
carcinomas with apocrine differentiation	5	5	0
invasive micropapillary carcinoma	6	3	3
invasive papillary carcinoma	8	2	6
carcinomas with medullary features	22	8	14
Other invasive carcinoma	5	0	5
